# No difference in patient‐reported outcomes or range of motion between ultracongruent and posterior stabilized total knee arthroplasty: A randomized controlled trial

**DOI:** 10.1002/jeo2.70043

**Published:** 2024-10-21

**Authors:** Fernando Macedo, João Lucas, Patrícia Cunha, Miguel Rocha, Rui Cerqueira, Tiago Basto, João Moura

**Affiliations:** ^1^ Orthopedics and Traumatolgy Department Hospital Senhora da Oliveira Guimarães Portugal

**Keywords:** anterior stabilized, posterior stabilized, TKA, TKR, ultracongruent insert

## Abstract

**Purpose:**

Ultracongruent (UC) inserts were designed to overcome potential issues with posterior stabilized (PS) inserts, including bone resection, post‐breakage, and patellar clunk syndrome. However, there remains a shortage of high‐quality studies directly comparing this insert design to the established PS Total Knee Arthroplasty (TKA). This prospective randomized controlled trial (RCT) aimed to compare clinical outcomes, range of motion, and complications of UC and PS TKA.

**Methods:**

Ninety six patients with primary knee osteoarthritis were randomly assigned to either the PS or the UC group. There were no significant differences between the groups regarding age, body mass index, sex, or Osteoarthritis grade. The measured outcomes were Knee Injury and Osteoarthritis Outcome Score (KOOS) and Range of Motion (ROM), recorded preoperatively and at 3 and 6 months postoperative. Both the patient and interviewer were blinded to the allocation group.

**Results:**

Eighty one patients were included in the analysis, with a mean follow‐up of 1.3 years. Both groups exhibited a gradual improvement in KOOS. Still, no statistically significant differences were observed between the groups during the follow‐up examinations at 3 or 6 months in KOOS or range of motion. No complication occurred in either group during the follow‐up period.

**Conclusion:**

Both designs demonstrated comparable positive outcomes, reinforcing the viability of UC designs as an alternative to the well‐established PS TKA.

**Levels of Evidence:**

Level l, randomized controlled trial.

**Abbreviations**
CR: cruciate‐retaining
KOOS: Knee Injury and Osteoarthritis Outcome Score
PRO: patient reported outcome
PS: posterior stabilized
RCT: randomized controlled trial
ROM: range of motion
TKA: total knee arthroplasty
UC: ultracongruent

## INTRODUCTION

In the archives of knee replacement history, posterior‐stabilized (PS) and cruciate‐retaining (CR) constructs have been the most popular tibial inserts. However, according to the American Joint Replacement Registry, a recent shift has been observed with the rise in popularity of ultra‐congruent (UC) designs, constituting 25% of the inserts utilized [7].

PS inserts are associated with some disadvantages, such as additional bone resection, breakage or dislocation of the post and patellar clunk syndrome [14]. The downside of the increased mechanical articulation in a PS design might become more pronounced in the future with the integration of new technologies like cementless fixation and highly crosslinked polyethylene, which may demonstrate reduced reliability in an environment characterized by increased mechanical stresses [13].

UC inserts were designed to prevent these drawbacks [15]. Nevertheless, UC inserts have many concerns, such as restricted femoral rotation, paradoxical anterior translation, and diminished sagittal tibial stability. These factors collectively contribute to altered knee kinematics and have raised apprehensions about potential adverse clinical outcomes [2, 6].

Despite the growing emphasis on ultra‐congruent inserts in arthroplasty literature and their increasing utilization in clinical practice, there remains a shortage of high‐quality studies directly comparing insert designs [2, 18]. Currently, there is no consensus in the literature regarding the superiority of either of these inserts, as highlighted by a recent meta‐analysis, which underscores the necessity for a more robust investigation [18].

The aim of this prospective randomized controlled trial (RCT) is to compare patient‐reported outcome (PRO) and range of motion (ROM) between UC and PS Total Knee Arthroplasty (TKA). It was hypothesized that PS inserts had better ROM, while there was no difference in PRO between both designs.

## METHODS

The study was performed in compliance with the Helsinki Declaration and was approved by the local ethics committee. All participants provided written and informed consent.

A randomized controlled trial was performed. All patients with primary osteoarthritis of the knee scheduled for a condylar TKA were screened. Patients who needed a higher constraint TKA were not included.

The functional outcome was measured by the Knee Injury and Osteoarthritis Outcome Score (KOOS) and knee ROM, which were measured using a 60‐cm goniometer with the patient in a supine position.

The exclusion criteria were inflammatory arthritis, posttraumatic arthritis, or a prior surgical procedure in the knee.

On the day of admission, one of two surgeons observed the patients, checked for exclusion criteria, and collected pre‐operative KOOS and knee ROM. The patients were allocated in a 1:1 ratio via computer‐generated randomization using Microsoft Excel 2016 (Microsoft Corporation) to either the UC insert or PS insert group. The allocation group was concealed from the patient and all personnel. The senior surgeon was informed of the allocation previously to surgery by electronic message.

**Figure 1:**
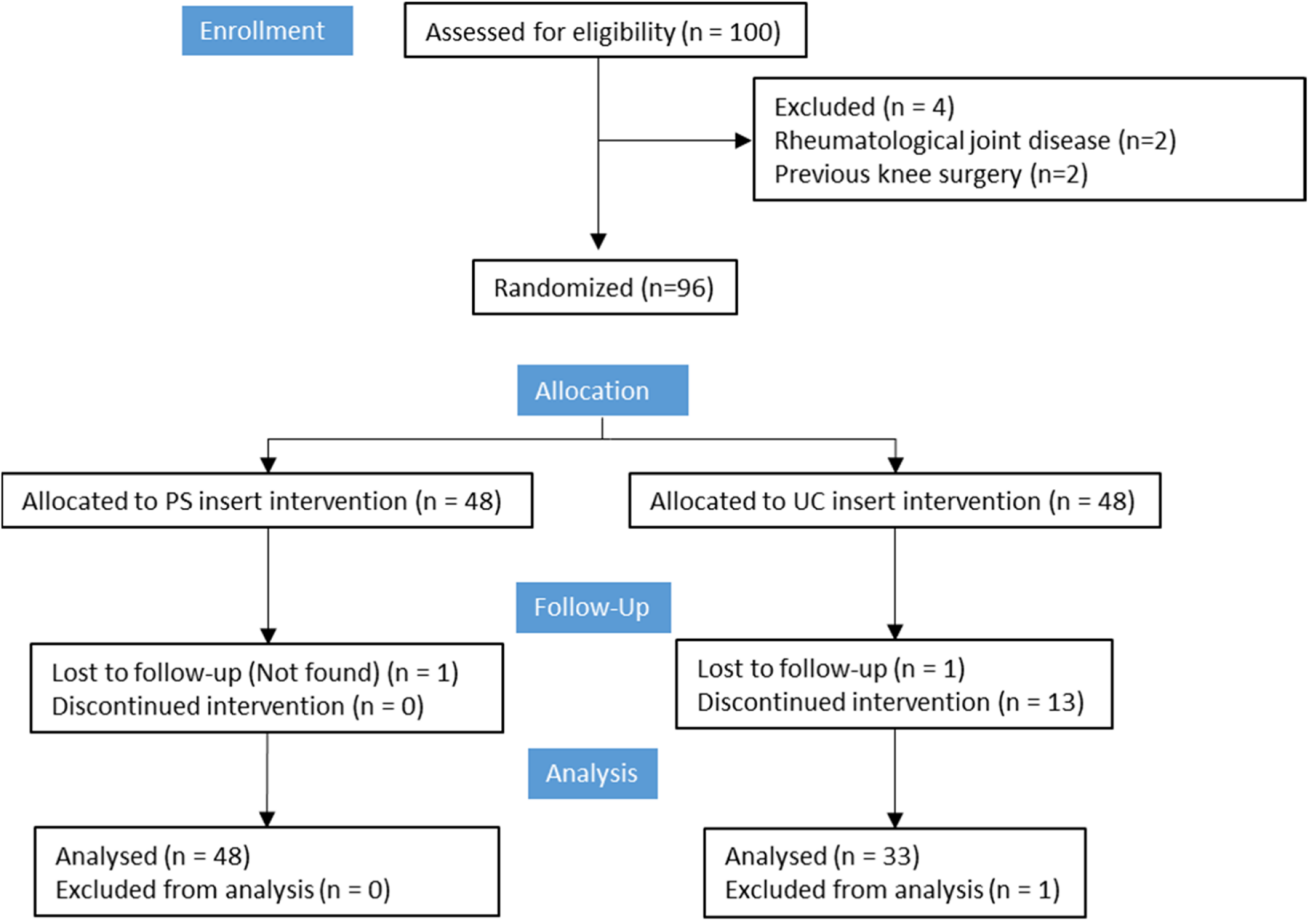
Consolidated standards of reporting trials (CONSORT) flow diagram.

Hundred patients were assessed for eligibility, and 96 patients were randomized. 48 were allocated with a UC TKA and 48 with a PS (Figure 1). Postoperative interviews were carried out by one of two residents, blinded to the patient allocation, with the patient also kept unaware of the inert used. These interviews took place at the 3‐month and 6‐month postoperative, involving the collection of ROM, KOOS assessments, clinical examination, and anteroposterior and lateral radiographs. Comprehensive recording of all complications was maintained throughout the study.

### Statistical analysis

Statistical analyses were performed using SPSS (version 26.0 for Windows; IBM), and significance was set at 0.05. Data were reported as means and standard deviation (SD) for continuous values and absolute frequencies for categorical values, respectively. Comparisons between groups were done using a paired *t*‐test for continuous values and a Chi‐square test for categorical values.

### Operative procedure

All patients underwent a cemented fixed‐bearing TKA (Multigen Plus, Lima Corporate) without patellar resurfacing. The surgeries were conducted using a tourniquet following a single antibiotic dose (cefazolin 2 g).

For the UC TKA, a standard femoral component was utilized, while additional box cuts were necessary for the PS TKA. All surgeries were performed using the same technique, with extramedullary alignment, by one of three senior surgeons specialized in knee surgery. A suction drain was uniformly applied in all cases and removed 24 h after surgery.

A standardized postoperative protocol was administered to all patients, encompassing pain control medication and physiotherapy, overseen by a nurse specialized in arthroplasty rehabilitation, who was blinded to the assigned surgical procedure. Patients were mobilized with partial weight‐bearing on the day following surgery and were instructed to continue using crutches for four weeks. At the 4‐week mark, a clinical and radiographic assessment was conducted, after which patients began complete weight‐bearing activities.

## RESULTS

Out of the 48 patients assigned to the UC group, the intervention was not performed in 13 cases due to incompatibility between the tibia and femur sizes. It is worth noting that, in the Multigen Plus model, the tibia size must be equal to or larger than the femur size for a UC insert to be possible.

One patient in the UC group was lost to follow‐up and did not participate in any of the postoperative interviews. Additionally, another patient in the UC group was excluded from the analysis due to confounding factors; specifically, the patient developed Lumbar Spinal Stenosis and subsequently underwent surgical decompression.

In the analysis, 48 patients were included in the PS group and 33 in the UC group. The age, sex, and body mass index distributions of the groups did not differ, as shown in Table 1.

**Table 1:** Demographic characteristics of the patients.*

Patient characteristics	PS group	UC group	*p*‐Value
Gender			0.78
Female	32	21	
Male	16	12	
Osteoarthritis grade^a^			
II	12	9	0.52
III	26	14	
IV	10	10	
Age at surgery (years)	72. ± 6.3	69.6 ± 6.4	0.08
BMI (kg/m^2^)	29.4 ± 4.5	30.7 ± 4.8	0.21

Abbreviations: BMI, body mass index; PS, posterior stabilized; UC, ultracongruent.

* Values are given as the frequencies for categorical variables and mean and standard deviation for quantitative variables.

^a^ Osteoarthritis grade according to Kellgren and Lawrence.

**Figure 2:**
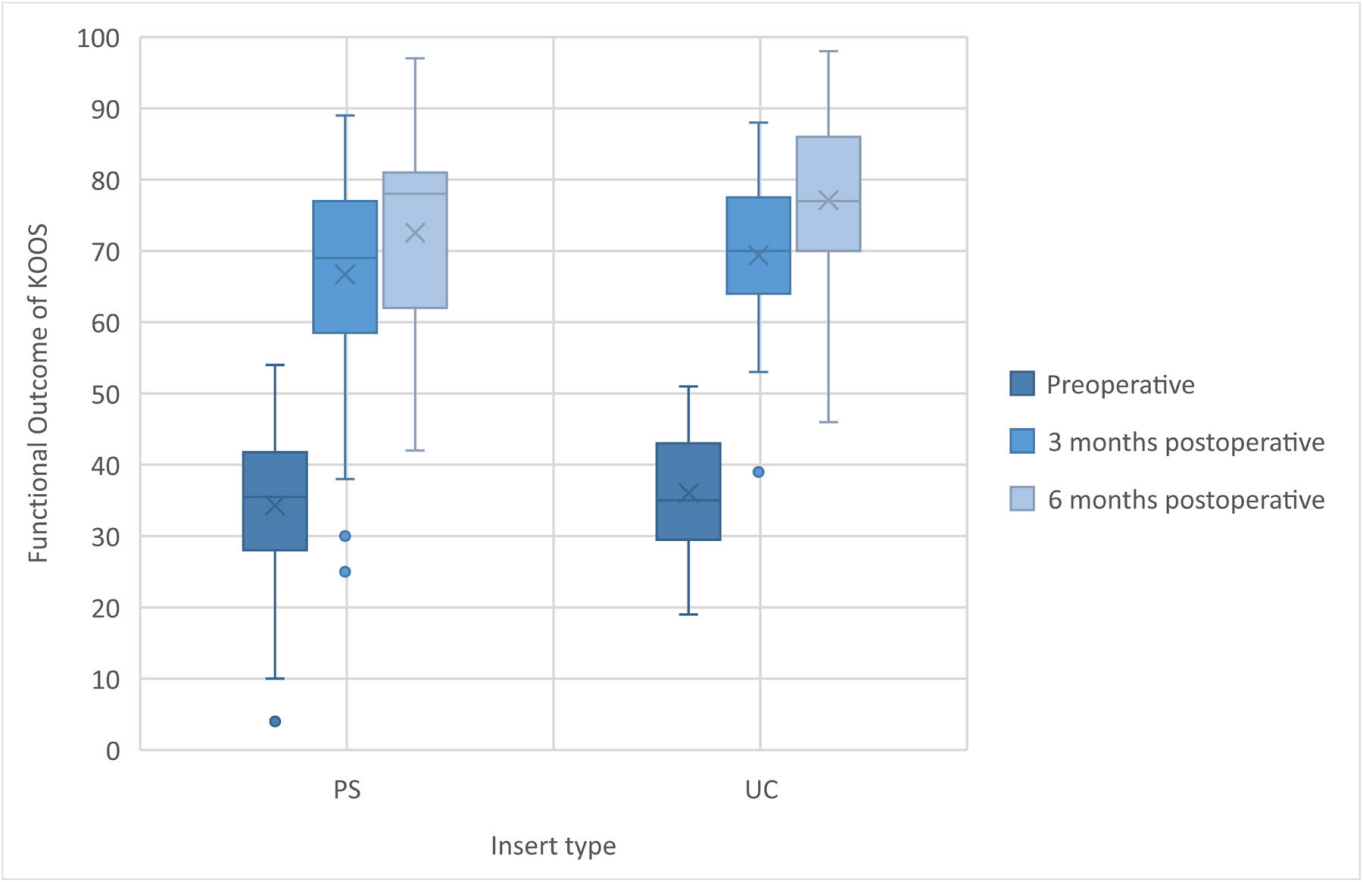
Preoperative and follow‐up KOOS (Knee Injury and Osteoarthritis Outcome Score) in both insert groups.

Both insert groups exhibited a gradual improvement in KOOS (Figure 2). Still, no statistically significant differences were observed between the groups during the follow‐up examinations at 3 or 6 months postoperative in KOOS (*p* value 0.37 and 0.12, respectively) or range of motion (*p* value 0.92 and 0.12). Across the subcategories of KOOS, no statistically significant differences were noted between the groups (Table 2). Notably, in the Sport and Recreation function section, the UC insert demonstrated a slightly better mean score (45) than the PS insert (35). However, this difference did not reach statistical significance (*p* value 0.06).

**Table 2:** Preoperative and postoperative functional outcomes.*

	Preoperative			3 Months postoperatively			6 Months postoperatively		
	PS	UC	*p*‐Value	PS	UC	*p*‐Value	PS	UC	*p*‐value
Flexion contracture (°)	5.9 ± 4.8	4.4 ± 2.5	0.09	5.6 ± 4.0	4.4 ± 2.8	0.15	3.1 ± 2.0	2.6 ± 2.2	0.306
Maximum flexion (°)	106.1 ± 12.1	107.4 ± 13.3	0.60	106.0 ± 11.5	105.1 ± 13.2	0.76	111.8 ± 10.0	106.9 ± 14.7	0.074
ROM (°)	100.2 ± 14.7	103.2 ± 14.0	0.356	100.4 ± 12.9	100.7 ± 14.1	0.92	108.7 ± 10.3	104.2 ± 15.2	0.117
KOOS	34.3 ± 11.5	36.0 ± 8.4	0.453	66.7 ± 14.1	69.4 ± 11.1	0.37	72.5 ± 13.7	77.1 ± 11.7	0.122
Symptoms	52.9 ± 22.0	57.9 ± 16.1	0.334	81.1 ± 16.1	84.6 ± 11.9	0.29	82.3 ± 17.3	86.6 ± 11.8	0.185
Pain	47.3 ± 16.4	52.2 ± 13.1	0.168	80.9 ± 14.4	83.5 ± 14.3	0.42	83.5 ± 18.6	88.9 ± 13.5	0.157
Activities of daily living	39.8 ± 14.9	43.4 ± 11.6	0.25	77.9 ± 16.8	80.1 ± 12.7	0.53	83.8 ± 15.9	89.0 ± 12.6	0.122
Sport and recreation function	5.6 ± 8.2	6.1 ± 7.4	0.81	30.9 ± 19.2	28.2 ± 21.1	0.55	34.8 ± 21.6	44.9 ± 24.9	0.06
Knee‐related quality of life	21.2 ± 14.5	21.9 ± 15.2	0.839	62.4 ± 21.6	70.3 ± 16.2	0.08	76.2 ± 18.9	77.9 ± 18.5	0.672

Abbreviations: FC, flexion contracture; KOOS, Knee Injury and Osteoarthritis Outcome Score; PS, posterior stabilized; ROM, range of motion; UC, ultracongruent.

* Values are given as the mean and standard deviation.

Regarding the range of motion, the PS group exhibited a 5° higher mean maximum flexion (112°) than the UC group (107°). However, this difference did not reach statistical significance (*p* value 0.074). There was no recorded complication reported in either group.

## DISCUSSION

The most important finding of this study was that there was no difference in range of motion and patient‐reported outcome (KOOS) between posterior‐stabilized and ultracongruent TKA designs. This aligns with the current literature and can reinforce the consideration of UC designs as a viable alternative to the well‐established PS options.

Over the past decade, modern CR or cruciate‐substituting bearings that are more conforming have emerged with the intent of substituting the PCL with a more pronounced anterior lip or through a conforming polyethylene articulation [17]. These modern bearings with greater conformity contrast traditional flat CR bearings, and clinical data has emerged with promising results [1, 8, 9, 10, 12, 16].

Kinematic studies demonstrated significantly more external rotation of the femur, less posterior femoral translation, greater tibial laxity in the sagittal plane, and less ROM in patients with UC inserts [2, 4]. However, despite the more natural knee kinematics in PS TKA, most RCTs have reported no difference in range of motion and clinical outcomes between PS and UC inserts. [1, 8, 9, 10, 11]. Bernasek et al. [3] found no difference in passive knee ROM, but when measuring in an unsupported squatting position, UC inserts exhibited a higher range of motion [3]. Conversely, Han et al. [5] reported PS inserts had better ROM. Akti et al. [1] studied isokinetic performance measured by peak extension torque and found no difference between inserts. Kim et al. [8] compared UC and PS inserts in 50 patients who underwent same‐day bilateral TKA, reporting more sagittal laxity with less roll‐back in the ultracongruent group but no difference in PRO, patient satisfaction, or joint perception. Lutzner reported 5‑year results of an RCT demonstrating similar good patient‐reported outcomes [11].

According to a recent meta‐analysis [18], the existing data indicates no clinical differences between PS and UC inserts. However, the authors of this review noted that among the Randomized Controlled Trials included, only one [8] met the criteria for “good quality.” Most RCT studies were rated poor due to numerous uncertainties associated with the protocols [18].

A notable limitation of this study is its relatively short follow‐up period of 6 months. Future studies with extended follow‐up durations are warranted to provide a more comprehensive understanding of the results and assess the long‐term viability of UC inserts.

Another limitation was the sample size and the disparity in frequency between the Posterior‐Stabilized and Ultra‐congruent groups due to incompatibility between femur and tibia sizes. Larger sample sizes are necessary to identify statistically significant differences in the KOOS subcategories. Only with such larger samples can we better understand the impact that the distinct knee kinematics between the two total knee arthroplasty designs may have on patients' daily lives.

Despite our acknowledged limitations, the strength of our study lies in its design as a randomized controlled trial, projected with a deliberate focus on minimizing bias. The author performed patient allocation using computer‐generated randomization, and the allocation group was kept concealed from both the patients and interviewers throughout the entire study duration. The senior surgeon was informed of the allocation before surgery via electronic message.

The clinical relevance of the present study is that ultracongruent TKA may be an alternative to posterior‐stabilized TKA with similar patient‐reported outcomes.

## CONCLUSION

Our study highlights that ultracongruent inserts exhibit comparable clinical outcomes and range of motion to posterior‐stabilized designs. This suggests that UC inserts can be safely considered a viable alternative to PS inserts in total knee arthroplasty.

## AUTHOR CONTRIBUTIONS

*Acquisition of data*: João Lucas, Patricia Cunha. *Analysis of data*: Miguel Rocha. *Conception*: Rui Cerqueira and Tiago Basto. *Design and revision*: João Moura.

## CONFLICT OF INTEREST STATEMENT

The authors declare no conflict of interest.

## ETHICS STATEMENT

The study has been performed in compliance with the Helsinki Declaration and has been approved by the local ethics committee. Every patient enrolled in the study has agreed and signed informed consent.

## Data Availability

Data that support the findings of this study are available on request from the corresponding author.
